# Front-loading of anatomy content has no effect on long-term anatomy knowledge retention among physical therapy students: a prospective cohort study

**DOI:** 10.1186/s12909-021-02925-z

**Published:** 2021-09-14

**Authors:** Amy H. Amabile, Kim Nixon-Cave, Larry J. Georgetti, Ashley C. Sims

**Affiliations:** 1 Department of Physical Therapy, Thomas Jefferson University, 901 Walnut Street, Philadelphia, PA 19107 USA; 2 University of Pittsburgh School of Health and Rehabilitation Sciences, Pittsburgh, PA USA; 3 NovaCare Rehabilitation, Cape May Courthouse, NJ USA; 4 St. Luke’s University Health Network, Bethlehem, PA USA

**Keywords:** Anatomy education, Spaced learning, Distributed learning, Massed learning, Long-term memory

## Abstract

**Background:**

Information learned over a longer period of time has been shown to result in better long-term knowledge retention than information learned over a shorter period of time. In order to address multiple curricular goals, the timing and spacing of anatomy content within the Doctor of Physical Therapy (DPT) program at our institution recently changed from a very spaced to a very compressed format. The purpose of the present study was to assess differences in anatomy knowledge retention that might have been impacted by this change. The research hypothesis was that students receiving spaced instruction would have significantly better anatomy knowledge retention than students receiving massed instruction.

**Methods:**

Participants consisted of two cohorts of DPT students that both received 45 contact hours of anatomy lecture and 90 contact hours of anatomy lab. The LONG cohort experienced anatomy through a lecture and lab taught over a 30-week, 2 semester period as separate courses. In contrast, the SHORT cohort took their anatomy lecture and lab concurrently over one 10-week semester. A pre-test was administered on the first day of their anatomy lecture course, and a post-test was administered to each cohort 18 months after completion of their last anatomy exam.

**Results:**

After controlling for age-related differences in the two groups, no significant differences in mean pre-test, post-test, or percentage improvement were found between cohorts (*p* = 0.516; 0.203; and 0.152, respectively).

**Conclusion:**

These findings refute the hypothesis and show that both spaced and massed instruction in these cohorts resulted in the same level of long-term anatomy knowledge retention.

## Background

There is a substantial body of research that shows that information learned over a longer period of time (spaced learning) results in better long-term retention than information learned over a shorter period of time (massed learning) [1–6]. Spaced learning (also known as “distributed learning”) can be described in terms of an increased amount of time spent on course delivery, and in terms of an increased number of retrieval/study episodes employed by a learner. There is a large amount of inconsistency in the use of the term within the literature, with a recent scoping review by Versteeg et al. [7] identifying over 70 different definitions of spaced learning among the health professions-related studies included in their analysis. This wide variation in terminology makes comparing spaced learning study results challenging, and requires that definitions of terms used within a study be clearly stated at the outset. The present research utilizes the definition coined by Kirkley [8] in the Encyclopedia of the Learning Sciences, which is as follows: “Distributed learning means that the material learned is distributed over a long period of time. .. [and] massed learning means that the material to be learned is provided within a short period of time.”

All types of semantic memories, including anatomical facts, will fade over time [1, 9–11], although periodic retrieval will increase the amount of material remembered [12, 13]. The greater knowledge retention typically seen with distributed versus massed learning is likely due to the fact that spacing of content delivery affords more retrieval/study opportunities to students. Another factor that favors spaced learning over massed learning is the decreased cognitive load seen with spacing of content. This is especially relevant in a course such as anatomy, which has been recognized as a subject that places high cognitive load demands on students [14–16]. The continual presentation of large amounts of new anatomy content on subsequent days is particularly difficult. This challenge is even greater when new material overlaps with or is similar to previously learned material, as this has been shown to interfere with consolidation of previously learned facts [17–19]. For example, the learning of wrist extensor muscle attachments and actions is made more difficult by the prior day’s presentation of the wrist flexor muscles. This happens because both groups of muscles have very similar names, attachments, and functions. In contrast, learning the attachments and actions of the rotator cuff muscles will not have the same potential to interfere with learning the wrist flexor muscles, because they are so different in naming, location, and function.

The Doctor of Physical Therapy (DPT) program at Thomas Jefferson University (TJU) utilizes a cohort-based, lock-step, nine semester curriculum. The structure of our program is similar to other DPT programs in the United States, in that it builds on a sequence of basic sciences and clinical skills-related coursework, preparing students for multiple terminal clinical affiliations before graduation.

Our program recently underwent a major change in timing and spacing of anatomy content, in order to help address several curricular goals aimed at increasing student success in both the didactic and clinical portions of the DPT curriculum. Prior to the change, anatomy had been taught in a spaced format as a separate 3-credit lecture course in fall of the first year, and a 3-credit gross anatomy laboratory course in the spring of the same academic year. In the revised curricular structure, anatomy lecture and lab were taught concurrently in a condensed 10-week semester which preceded all other basic sciences and clinical courses.

This change allowed for coverage of all anatomy content before initiation of any clinical didactic coursework, with an intent to elevate students’ subsequent performance in Biomechanics, Therapeutic Interventions, and Physical Therapy Examination. Based on prior research on spaced versus massed learning, however, there was a concern that this front-loading of anatomy might lead to decreased anatomy knowledge retention in the long-term. Long-term retention of anatomical facts is crucial not only to succeeding in later coursework within a DPT program, but also, arguably, to succeeding in physical therapy practice.

### Study goal, conceptual framework, and research hypothesis

The goal of the present study was to assess the impact of spacing changes in course delivery on anatomy knowledge retention in two cohorts of DPT students. Our conceptual framework recognized a multitude of factors affecting DPT student success in anatomy and other coursework. These include both cognitive variables such as undergraduate grade point average (GPA) and standardized test scores, as well as non-cognitive variables such as emotional intelligence and task coping abilities [20]. Spacing of content delivery, however, is one of the main success factors that we are able to control as educators. Given the ample evidence in the literature that spacing can affect learning outcomes, we felt it merited specific attention, and that it was of wide interest to DPT and other health sciences educators. This led to our present study design which had anatomy knowledge retention as the dependent variable, with a primary independent variable of participation in either a spaced or massed course delivery format. Additional independent variables, such as student age and non-traditional student status, were also considered for their potential role as covariates within the study. Our research hypothesis was that students receiving spaced instruction would have significantly better long-term anatomy knowledge retention than students receiving massed instruction.

## Methods

### Participants

This study utilized a prospective, cohort, pre-test post-test design with a convenience sample of DPT students. Participants consisted of two cohorts of students who both participated in 45 contact hours of anatomy lecture and 90 contact hours of anatomy lab during their first year as DPT students. The sole exclusion criteria was prior participation in an anatomy course within a DPT program. The LONG cohort consisted of students who participated in a 15-week Advanced Human Anatomy Lecture in the fall semester of their first year, and the 15-week Advanced Human Anatomy Lab in the spring semester of their first year. Of 61 students eligible to be in the LONG cohort, 40 consented to participate in the study and returned 18 months later to take the post-test. Of these 40 students, 2 had repeated anatomy within a DPT program and were therefore excluded from the study, resulting in a final LONG sample size of 38 students. The SHORT cohort consisted of students who participated in an Advanced Human Anatomy Lecture and Lab taught concurrently over a 10-week pre-fall semester in their first year as DPT students. Of 66 students eligible to be in the SHORT cohort, 34 consented to participate in the study and returned 18 months later to take the post-test, for a final SHORT sample size of 34 students.

### Procedures

Prior exemption from Institutional Review Board review was obtained from the TJU Office of Human Research (OHR) before initiation of data collection. The OHR is tasked with ensuring compliance with all federal and state laws pertaining to maintenance of ethical standards and safety within research projects involving human subjects. All students from both the LONG and SHORT cohort completed the study pre-test of baseline anatomy knowledge on the first day of the anatomy lecture course. Pre-test questions were chosen from the bank of course exam questions, and this assessment was routinely administered on the first day of class in order to customize course content to each cohort’s strengths and weaknesses. The pre-test consisted of 50 multiple choice questions covering basic anatomy knowledge in the cognitive domain of learning at a Bloom’s Taxonomy [21] level one of knowledge. The test consisted of 10 questions each from the 5 following regional anatomy knowledge subdomains: upper extremity, lower extremity, spine and back, thorax, and abdomen and pelvis.

Eighteen months after the completion of their final anatomy exam, students were invited to participate in the post-test, which was identical to the pre-test. This time frame was chosen because it is consistent with the definition of long-term memory established in the literature [2, 22], and because it reflected the practical needs of students to recall anatomy knowledge during later coursework and clinical affiliations. Students were allowed 50 min to complete both the pre-test and the post-test. The pre-test was administered using SofTest (Examsoft Worldwide, Dallas, TX) testing software on the students’ iPads; and, for logistical reasons, the post-test was administered using a paper exam. Student data, including DPT cumulative GPA at the time of the post-test, undergraduate GPA, accelerated degree status, non-traditional student status, and timing of undergraduate anatomy, were gathered and entered into data analysis to control for their impact as covariates on group means.

### Student background, course instruction, and content

All students within our DPT program have taken the same undergraduate prerequisites related primarily to basic sciences, and have met or exceeded a minimum cumulative undergraduate GPA of 3.0/4.0 points. Other factors, such as participation in service activities, and performance during face-to-face interviews, are also considered in admissions decisions. All first year DPT students hold a bachelor’s degree, except for a small number admitted through an accelerated degree program. These accelerated programs are relatively common in American higher education, and allow admission from select undergraduate institutions into the first year of physical therapy, occupational therapy, or medical school, after the third year of the student’s undergraduate program. Additionally, a minority of students admitted to the DPT program are considered “non-traditional,” in that they are entering a graduate program one or more years after completion of their undergraduate degree.

Course instruction was provided by the same faculty in the lecture portion, and primarily the same faculty for the lab portion for both cohorts. Content of both the lecture and lab portions differed only minimally between the cohorts, and consisted of minor changes to lectures and to lab dissection guides. Because of the short time frame, however, the SHORT cohort had content presented in 3 units with 3 sets of lecture and lab exams; while the LONG cohort had content presented in 4 units with 4 sets of lecture and lab exams.

The lecture portion consisted of 45 largely didactic contact hours, and the lab portion consisted of 90 contact hours of traditional cadaver lab dissection. When taught concurrently (for the SHORT cohort) lecture and lab content were synchronized. The order of topics for both cohorts was as follows: course introduction, peripheral nervous system, spine and back, lower limb, upper limb, thorax, abdomen, pelvis, head/neck and embryology.

One important difference between the cohorts was the number and types of non-anatomy courses taken concurrently with anatomy. The LONG cohort received anatomy instruction over two semesters with a total of 14 to 16 credits in each semester. Classes taught concurrently with anatomy included Biomechanics, Physical Therapy Exam, and Therapeutic Interventions. The SHORT cohort received anatomy instruction in a 10-week semester where students were registered for a total of 11 credits. No clinical courses, however, were taught concurrently with anatomy for the SHORT cohort. Although the ratio of credits per week is approximately the same for both groups, the impact of this difference is unknown. For example, biomechanical content taught concurrently with anatomy may have increased overall cognitive load for the LONG cohort. Yet it also may have reinforced anatomy content through the simultaneous application of anatomy to the learning of biomechanical content.

### Statistical analysis

Data were analyzed using SPSS version 25 (Armonk, NY) for Windows. Kolmogrov-Smirnov tests showed some data were normally distributed and some were not. However, sample size parameters [23] were satisfied for all variables so parametric tests were used for all data analysis. Chi square tests were used to measure association between categorical variables for the two groups, and point biserial correlations were used to compare categorical variables with continuous ones. Student’s t-test was used to compare mean post-test scores between the two cohorts and ANCOVA tests were used to control for the impact of potential covariates. Statistical significance was defined as *p* < 0.05.

## Results

Participant characteristics are detailed in Table 1. The cohorts were statistically identical in composition except for significant differences in age, numbers of non-traditional students, and years between undergraduate anatomy and anatomy in the DPT program.

**Table 1:** Participant demographics and characteristics

Variable	Mean (SD) LONG (***n*** = 38)	Mean (SD) SHORT (***n*** = 34)	***p-value***
Age	24.47 (1.22)	25.85 (2.97)	*0.015*
Sex - female/male	25/13	23/11	0.867
Non-Traditional	5	14	*0.007*
3 + 3 Students	12	5	0.092
UG Cumulative GPA	3.66 (0.18)	3.62 (0.27)	0.480
UG Science GPA	3.53 (0.27)	3.51 (0.30)	0.745
Years Between UG and DPT Anatomy	1.97 (1.44)	3.09 (1.99)	*0.008*
Cumulative DPT GPA at Post Test	3.62 (0.20)	3.58 (0.26)	0.428

*UG* undergraduate, *DPT* Doctor of Physical Therapy; 3 + 3 refers to accelerated degree students (3 years UG + 3 years DPT); Non-Traditional refers to students admitted to program with >/= one-year elapsed since completion of UG degree

Final course grades, cumulative DPT GPA at post-test, mean pre/post-test scores and improvement factor ((post-test score – pre-test score)/pre-test score) for both cohorts are shown in Table 2. Student’s t-tests for independent samples showed no statistically significant differences in the course grades, cumulative GPA, pre-test, post-test, or improvement factor between the groups. ANCOVA tests for a dependent variable of improvement factor were performed for the potential covariates of age, sex, non-traditional student status, and years elapsed between UG and DPT anatomy. After adjustment for these variables, there was still no statistically significant difference in improvement factor between the two cohorts. When all participants were combined for analysis, there was no correlation found between improvement factor and sex, age, non-traditional status, accelerated degree program participation, and undergraduate GPA. Pre-test score was found to be strongly, negatively correlated with improvement factor (*r* = − 0.75; *p* < .0005).

**Table 2:** Mean differences in test scores, course grades, and cumulative GPA between LONG and SHORT cohorts

Variable	Mean (SD) LONG (***n*** = 38)	Mean (SD) SHORT (***n*** = 34)	***p-value***
Course Grade Lecture	90.12 (0.09)	90.89 (0.07)	*0.596*
Course Grade Lab	93.39 (0.04)	91.91 (0.06)	*0.123*
Cumulative DPT GPA at Post-Test	3.62 (0.20)	3.58 (0.26)	0.428
Pre-Test %	33 (8)	32 (8)	*0.516*
Post-Test %	50 (10)	53 (9)	*0.203*
Difference	17 (11)	21 (11)	*0.124*
Improvement Factor	60 (46)	77 (53)	*0.152*

Note: Improvement Factor = ((post-test score – pre-test score)/pre-test score)

## Discussion

The results of the present research refute our hypothesis that students receiving spaced instruction would have significantly better long-term anatomy knowledge retention than students receiving massed instruction. The two cohorts had statistically equal pre-test, post-test, and improvement factor scores, even when controlling for significant differences in participant characteristics. While these results apply to our two cohorts of DPT students, they cannot necessarily be extrapolated to cases of spaced versus massed course delivery with other samples and in other circumstances, due to potential confounding factors described below.

Our results contrast with other studies showing spaced learning is more beneficial in long-term memory retention [3, 4, 24–27]. Spaced learning allows for more frequent and temporally distributed retrieval opportunities, which have been shown to increase long-term knowledge retention [4, 28–30]. Increased spacing also decreases cognitive load by decreasing the number of elements being processed by working memory in a given time period [15].

We have identified only a limited number of studies where massed learning proved superior to spaced course delivery. The study designs, time frames, target population, and types of learning vary greatly, and cannot be easily compared to one another, nor to studies measuring acquisition of anatomical facts. Their existence, however, supports the idea that in certain circumstances learning can be potentiated through the use of massed instruction and fewer retrieval episodes, and this topic merits further research. Circumstances where massed learning may be more beneficial tend to involve motor learning and include: patient acquisition of upper limb motor skills after stroke with the use of constraint induced movement therapy delivered in a massed format [31]; improvement in the ability to read radiographic images through massed practice sessions believed to improve eye tracking abilities [32]; and improvement in objective and subjective vocal function through vocal rehabilitation exercises delivered in a massed format to patients with dysphonia [33].

While our main findings were surprising, other known patterns of student test behavior were borne out by our results. A strong, negative correlation was found between pre-test scores and improvement percentage, reflecting the known phenomenon that participants with lower baseline scores will generally improve more than those with higher baseline scores [34, 35]. Similarly the fact that students’ mean post-test scores, while increasing substantially compared with the pre-test scores, were still quite low, is in agreement with previous research showing significant loss of basic science knowledge over time [9, 36, 37]. This type of knowledge will inevitably be forgotten over a period of months and years following the last study session [38].

A search of the literature found no other anatomy-related studies on spacing and knowledge retention that utilized a retention interval length (i.e. the time between the final study session and the post-test) commensurate to the one used in the present research. A limited amount of research has, however, been performed assessing variance in anatomy knowledge retention using shorter retention intervals. For example, Dobson et al. [1] assessed anatomy recall within an undergraduate student cohort, using a variety of retrieval strategies and time between study/retrieval sessions. They did find that distributed practice benefitted their purported long-term (28-day) retention significantly more than massed practice. The short retention interval used in this research does, however, limit the external validity of their results for DPT and medical students, for whom anatomy recall is required over a period of years during school and beyond.

In addition to the length of anatomy instruction, the LONG and SHORT cohorts experienced other differences in their overall DPT curricula, which may have affected the present findings. Although the general sequence of courses remained the same for both cohorts, the LONG cohort had clinical affiliations that began earlier in their curriculum, at the end of their first year of didactic instruction, while the SHORT cohort’s affiliations were set to begin at the end of their second year of didactic instruction. Thus, at the time of the post-test, the LONG cohort had already completed three clinical affiliations (for a total of 26 weeks), while the SHORT cohort had not yet participated in any full-time clinical affiliations at the time of their post-test. An increasing body of research supports the concept that clinical experience can actually interfere with knowledge retention, and that the development of clinical reasoning requires a certain amount of forgetting of the details of underlying basic science and medical knowledge. This phenomenon has been studied among medical students, residents, and physicians and is known as the “intermediate effect” [39]. According to this theory, their 26 weeks of clinical affiliations may have given the LONG cohort relative expert status. Once practitioners are experts, recall of facts is inevitably attenuated as they start to develop more clinical reasoning skills that allow them to understand a problem at a deeper level and to incorporate the use of causal models or illness scripts in patient treatments. Conversely, the SHORT cohort, having finished virtually all of their didactic coursework but lacking in significant clinical experience, can be considered to have intermediate expertise, and according to the intermediate effect, these students should have the best recall of facts [39, 40].

The potential impact on the SHORT cohort of taking the anatomy lecture and lab concurrently is another important difference between the curricula experienced by the two cohorts. This may have had a positive effect on their test scores, because of the potential benefits to students who are kinesthetic learners. Although the importance of learning styles has been disputed [41], some students do identify as kinesthetic learners and express a preference for learning opportunities that combine didactic and kinesthetic approaches [42].

### Limitations

Sample size limitations related to our use of a convenience sample may have affected the lack of significant differences in anatomy knowledge retention seen among our two cohorts. Our results did trend in favor of the SHORT cohort, but additional participants could have strengthened this into a significant difference, or even potentially have reversed it. Using just these two cohorts, however, allowed for the control of most other differences in instructional delivery that occur inevitably over time and, we felt, increased the overall quality of our sampling.

As discussed above, the change in anatomy timing was just one component of a major curricular change that impacted multiple aspects of the DPT program at our university. Although anatomy content presented was virtually identical for both cohorts, other differences in their educational experiences could not be avoided. Specifically, the concurrent lecture and lab experienced by the SHORT cohort, and the differential timing between the cohorts of participation in clinical affiliations, may have affected post-test performance for both of our cohorts.

## Conclusions

The results of the present research show that massed learning was not detrimental to long-term retention of anatomy knowledge among these two cohorts of DPT students. These findings may have implications not just for anatomy instruction, but potentially for any content-heavy, basic sciences material taught within a health sciences curriculum. A small sample size, as well as other factors, limit the generalizability of our findings. In particular, the possible influence of clinical experience and concurrent lecture and lab content delivery should be considered in any future research designed to assess this phenomenon.

## Data Availability

The datasets used and analyzed during the current study are available from the corresponding author on reasonable request.

## Abbreviations

DPT: Doctor of Physical Therapy
LONG: Refers to cohort receiving two 15-week semesters of anatomy
SHORT: Refers to cohort receiving one 10-week semester of anatomy
GPA: Grade point average
UG: Undergraduate
